# Excessive adiposity, metabolic health, and risks for genital human papillomavirus infection in adult women: a population-based cross-sectional study

**DOI:** 10.1186/s40608-015-0071-3

**Published:** 2015-10-01

**Authors:** Su-Hsun Liu, Hsin-Jen Chen, Tsung-Han Hsieh, Jih-Chang Chen, Yhu-Chering Huang

**Affiliations:** College of Medicine, Chang Gung University, Wen Hwa 1st Rd., Gueishan District, Taoyuan City, 333 Taiwan; Department of Family Medicine, Chang Gung Memorial Hospital at Linkou, 5 Fuhsin Street, Gueishan District, Taoyuan City, 333 Taiwan; Department of Public Health, School of Medicine, National Yang Ming University, No.155, Sec.2, Li Nong Street, Taipei, 112 Taiwan; Department of Emergency Medicine, Chang Gung Memorial Hospital at Linkou, 5 Fuhsin Street, Gueishan District, Taoyuan City, 333 Taiwan; Department of Pediatrics, Chang Gung Memorial Hospital at Linkou, 5 Fuhsin Street, Gueishan District, Taoyuan City, 333 Taiwan

**Keywords:** Human papillomavirus infection, Obesity, Cervical cancer, Metabolic health

## Abstract

**Background:**

The role of excessive adiposity or its metabolic consequences in persistent HPV infection among general adult women remains unknown.

**Methods:**

Using data from the National Health and Nutrition Examination Survey (NHANES) in 2003–2010, we compared adult women’s likelihood for any- or high-risk (HR) type HPV infection by degrees of excessive adiposity and metabolic health status.

**Results:**

Any-type (41.1 % vs. 44.9 %, *P* = 0.045) or HR-type HPV prevalence (21.9 % vs. 25.4 %, *P* = 0.055) was comparable in women aged 20–59 years with or without central obesity. After adjusting for age, socioeconomic indicators, and lifetime sexual risks, centrally-obese women barely showed a different likelihood for any-type (aPR [adjusted prevalence ratio] = 0.91, *P* = 0.03) or HR-HPV infection (aPR = 0.92, *P* = 0.279). However, obesity (aPR = 0.76, *P* = 0.017) or centrally-obesity (aPR = 0.72, *P* = 0.003) was negatively correlated with HR-HPV infection in women reporting an early sex debut (<16 years; P for interaction <0.05). In the fasting subpopulation, obesity (aPR = 0.77, *P* = 0.016) or metabolically unhealthy obesity (aPR = 0.69, *P* = 0.018) was significantly correlated with a 23 % or 31 % reduced prevalence of HR-HPV infection.

**Discussion:**

In contrary to findings for the general population, HR-HPV prevalence was decreased in a subgroup of women with obesity or central obesity. Possible explanations for such heterogeneity included less risky sexual behaviors, an altered immune milieu that promoted viral clearance, and increased access to healthcare resources due to other obesity-related co-morbidities in this subpopulation.

**Conclusions:**

Obesity or central obesity was not significantly associated with prevalent any-type or HR-type HPV infection among adult women in general. However, in certain subpopulations, excessive adiposity or its relevant metabolic dysfunction was negatively associated with HR-HPV infection.

**Electronic supplementary material:**

The online version of this article (doi:10.1186/s40608-015-0071-3) contains supplementary material, which is available to authorized users.

## Background

Globally, cervical cancer has ranked the fourth most common cancer in women and is responsible for 7.5 % of all cancer mortality for females in 2012 [1]. While most genital human papillomavirus (HPV) infections are believed to be transient, [2] for a small at-risk subpopulation, high risk (HR) or oncogenic type HPV infections can persist and eventually lead to carcinogenesis at the cervix, anus, oropharynx and other anatomical sites 10–20 years later [1, 3]. Despite continuous epidemiological and clinical research efforts, host factors for viral clearance or persistence remain largely unknown [2]. Lessons learned from immunocompromised women, such as those living with human immunodeficiency virus (HIV), have suggested that host immunity may play a key role in the clearance or reactivation of HPV [4, 5].

Not only healthcare-associated infections, such as surgical site infection, or aspiration pneumonia among hospitalized patients, [6] an accumulating body of evidence has suggested that obesity may predispose individuals to an increased risk for acute and chronic community-acquired infections, such as H1N1 influenza virus, [7] herpes simplex virus type 1 (HSV-1) [8] or HPV. [9] Comparing healthy obese (BMI ≥ 25) with lean (BMI < 25) subjects, Nieman and colleagues found that obesity was associated with differences in numbers and chemotactic responses in subsets of immune cells [10]. Despite this growing body of evidence showing that a pro-inflammatory state is often associated with excess adiposity and its metabolic consequences, [11] it remains unclear whether women of excessive adiposity have an altered immune status affecting their likelihood for HPV persistence.

Previously, we and other colleagues have shown that obesity or central obesity did not predict the incidence or regression of HPV DNA detection among a cohort of U.S. perimenopausal women (aged 35–60) over a median follow-up period of 18 months [12]. As BMI may not optimally reflect an individual’s visceral adiposity, [13] studies have suggested alternative indices, such as waist circumference (WC), [14] waist-to-height ratio (WHtR), [15] or waist-to-hip ratio (WHR) [16] to better correlate with the dysmetabolic state or the degree of low grade inflammation. Our early cohort study did not address the potential health effects of excess adiposity on HPV infection among adult women in general. Nor did it identify any subgroups of women who may have differential risks for persistent HPV infections.

Recently, metabolic health has gained an increasing attention to its potential mediating role in cardiovascular diseases [17] and cancers [18]. Yet, few studies have specifically investigated on metabolic health in obesity-related infection risks. The previous prospective study did not specifically examine the role of women’s metabolic health in HPV infection due to the lack of relevant measurements [12]. Assuming that HPV prevalence parallels women’s risk for chronic, persistent HPV infection at the population level, [19] we aimed to compare HPV prevalence between women with and without excessive adiposity, as determined by a large waist (>88 cm, central obesity) or by BMI (≥30 Kg/m^2^), using data from the continuous National Health and Nutrition Exam Survey (NHANES) in 2003–2010. Also, we sought to explore whether women’s risk for persistent HPV infection differed by metabolic health status in the fasting subgroup. In particular, we attempted to identify subpopulations of women with susceptibility or resistance to HPV persistence if applicable.

## Methods

### Study design and population

The National Center for Health Statistics (NCHS) has obtained ethical approval from NCHS Research Ethics Review Board before each survey cycle [20]. Details regarding the survey design, contents of questionnaire, and data collection of the continuous NHANES program can be accessed online [21]. In brief, adults aged 20 or older were interviewed during a household visit and examined at the mobile examination center (MEC) where specimens were also collected. Comprehensive demographic, socioeconomic and behavioral information were documented at the interview. For the subgroup analysis, we included women whose anthropometric measures and fasting laboratory findings were available for characterizing a subject’s metabolic health status. Only women attending the morning session and who had fasted ≥ 8 h were included for the subgroup analysis. Self-reported pregnancy status at the time of the exam was further confirmed by results of urine pregnancy test. As the current study involved secondary analysis of publicly available, de-identified data, the institutional review boards of the Chang Gung Memorial Hospital at Linkou has reviewed the analytic plan and waived the consenting requirement (No.102–0389B).

### Data collection

In 2003–2004, HPV DNA extracted from women’s self-collected vaginal swabs were tested for the presence of 37 HPV types using the Roche prototype line blot assay, which was replaced by the Roche Linear Array (LA) kit since 2005 [21]. Residual DNA extracts from NHANES 2003–2004 were thus retyped using the LA kit to allow for data comparison and trend analysis [22]. The current analysis was based on the LA genotyping results, and a swab result was excluded when the swab was negative for human β-globin. Any-type HPV positivity was defined by at least one positive out of 37 genotypes whereas oncogenic type positivity was determined by positive findings in the following 14 types: 16, 18, 31, 33, 35, 39, 45, 51, 52, 56, 58, 59, 66, and 68 [21].

### Definitions for excess adiposity

Obesity was defined based on WHO classification (BMI ≥ 30 kg/m^2^) [23] whereas central obesity was determined based on the National Cholesterol Education Program’s Adult Treatment Panel III report (ATP III, >88 cm for women) [24]. A cut-off value of 0.6 was employed for using waist-to-height ratio (WHtR) to characterize women of a large waist [15].

### Definitions for metabolic health

For the fasting subgroup, the ATP III criteria was also adopted for defining metabolic syndrome (MetS) according to the presence of the following metabolic risk factors, including central obesity; the diagnosis of or the current use of prescription drugs for hypertension (blood pressure ≥130/85 mmHg) or diabetes (fasting glucose ≥ 100 mg/dL); a dyslipidemic profile (triglyceride [TG] ≥ 150 mg/dL or high-density cholesterol [HDL] <50 mg/dL for women). A previously published definition was applied to defining individual metabolic health status as healthy (none or one of metabolic risk factors) or unhealthy (two or more metabolic risk factors) such that participants were further grouped into one of the four metabolic-obese phenotypes based on their obesity and metabolic health status- metabolically healthy and nonobese (MHNO), metabolically healthy but obese (MHO), metabolically unhealthy nonobese (MUNO) and metabolically unhealthy obese (MUO) [25]. Different criteria for central obesity (WHO recommendation: 80 cm for Europid or Non-Hispanic White women) [26], hypertriglyceridemia (177 mg/dL or 2.0 mmol/L) and for MetS (The International Diabetes Federation [IDF] consensus) [27] were considered in separate sensitivity analyses.

### Other covariates

A participant’s race or ethnicity identity was grouped into one of the following categories: non-Hispanic White, non-Hispanic Black, and others including Mexican American. Women were also classified as being married, single, or living with a partner according to self-reported relationship at the time of the interview. The question about health insurance coverage did not discern about public or private insurance providers. Information regarding other cardio-metabolic risk factors was based on self-report, including the medical history of hypertension and diabetes. To avoid surveillance bias, we did not consider a participant’s average blood pressure measurement (systolic >140 mg or diastolic >85 mg) or plasma levels of fasting glucose (>100 mg/dL, if available) at the time of the survey but only her self-reported clinical diagnosis and prescribed medication usage to infer her medical conditions.

Behavioral risk factors included: lifetime smoking ≥100 pieces, alcohol use >12 drinks in the previous 12 months; sexual exposure history as categorized by numbers of cumulative and recent sexual partners: low (lifetime sexual partners <5), moderate (lifetime sexual partners ≥ 5 and recent sexual partners <5 over the previous 12 months), and high risk (both lifetime and recent sex partners ≥ 5); self-reported use of condom (not always versus always) in the previous 30 days (in 2003–2004) or in the previous 12 months (in 2005–2010); current use of oral contraceptive use or not.

### Statistical analysis

Applying sampling weights to account for the multi-level, stratified, probability sampling and non-response rates in the NHANES, [22] we calculated population-based prevalence estimates of any-type HPV infection for the overall study populations and by age groups, race or ethnicity, survey years, and other individual characteristics. We compared prevalence of any-type and HR-type HPV by nominal covariates using design-based Chi-squared statistics. Due to the high prevalence of HPV infection, we applied Poisson regression models to compare adjusted prevalence ratios [28]. We also confirmed model results with those obtained by use of negative binomial models to ensure the variance assumption was not violated in Poisson models. In addition to stratified analysis, we further evaluated the following explanatory variables for a potential modulating effect on the adiposity-HPV association: age (<35 vs. 35 years or older); ethnicity (non-Hispanic white vs. non-Hispanic black vs. others); education (high school or fewer vs. college or higher); age at sexual debut (<16 vs. 16 years or older); high cumulative sexual exposure (<5 vs. 5 lifetime sex partners or more). All analysis was performed in Stata 13 with a two-tailed significance level of 0.05 [29].

## Results

In 2003–2010, 16,729 adults aged 20 years or older completed measurements of blood pressure, body weight and height, and waist circumference (Additional file 1: Figure S1). Women aged 20–59 years were asked to provide a vaginal swab, from which the LA genotyping results for HPV DNA testing were available from 5372 individuals. Among 4172 women who were included in the primary analysis (Additional file 1: Figure S1), there was no significant temporal change in any-type or HR-type HPV prevalence across the eight survey years (*P* = 0.143 and *P* = 0.853, respectively, Table 1).

**Table 1:** Weighted prevalence of any-type and high-risk type HPV by selected characteristics of women aged 20–59 (*N* = 4172) in NHANES 2003–2010

Participant characteristics	Any-type HPV		High-risk type HPV	
	%	*p*-value^a^	%	*p*-value^a^
Overall	42.6 %		23.3 %	
Survey year				
2003–2004	47.2 %	0.143	23.5 %	0.853
2005–2006	40.3 %		24.7 %	
2007–2007	41.5 %		22.7 %	
2009–2010	42.4 %		22.7 %	
Adiposity metrics				
Central obesity: waist > 88 cm	41.1 %	0.045	21.9 %	0.055
waist < = 88 cm	44.9 %		25.4 %	
Obesity: BMI > = 30 Kg/m2	41.7 %	0.451	22.0 %	0.191
BMI < 30 Kg/m2	43.2 %		24.0 %	
Waist-to-height ratio: ≥ 0.6	42.5 %	0.884	22.2 %	0.209
< 0.6	42.7 %		24.0 %	
BMI, Kg/m^2^				
18.5–25	42.4 %	0.360	23.9 %	0.345
25–30	44.2 %		24.3 %	
30–35	39.7 %		20.3 %	
≥ 35	43.6 %		23.6 %	
Socio-demographics				
Age, years				
20–34	51.8 %	<0.001	33.9 %	<0.001
35–49	40.1 %		20.2 %	
50–59	35.0 %		14.8 %	
Race/ethnicity				
Non-Hispanic White	39.6 %	<0.001	21.8 %	<0.001
Non-Hispanic Black	59.9 %		33.0 %	
Mexican American/others	43.3 %		23.1 %	
Education ≥ College degree	33.0 %	<0.001	15.6 %	<0.001
High school or less	46.4 %		26.3 %	
Marital status				
Married	30.0 %	<0.001	13.9 %	<0.001
Single	59.2 %		35.7 %	
Living with a partner	59.0 %		35.5 %	
Health insurance coverage^b^: yes	40.0 %	<0.001	21.6 %	<0.001
no	54.2 %		30.7 %	
Behavioral risks				
Hypertension history^c^: yes	41.1 %	0.419	20.2 %	0.021
no	43.0 %		24.2 %	
Diabetes history^d^: yes	46.5 %	0.285	19.0 %	0.209
no	42.5 %		23.7 %	
Lifetime smoking: ≥ 100 cigarettes	49.4 %	<0.001	26.4 %	0.004
< 100 cigarettes	37.3 %		20.9 %	
Last 12 months had ≥ 12 alcohol drinks^e^	44.4 %	0.001	25.0 %	<0.001
< 12 alcohol drinks	37.7 %		18.7 %	
Age at sex debut > =16	39.3 %	<0.001	21.6 %	<0.001
<16	52.8 %		28.6 %	
Number of lifetime sex partners > =5^f^	54.5 %	<0.001	31.3 %	<0.001
< 5	28.2 %		14.1 %	
Condom use: not always^g^	50.6 %	<0.001	32.0 %	<0.001
Always	37.7 %		19.4 %	
Use of birth control pills: currently^h^	45.7 %	0.173	29.2 %	0.003
Not currently	42.3 %		22.5 %	

*BMI* body mass index, *SE* standard error

^a^ *P*-value for survey-based Chi-squared test

^b^Six women had a missing value

^c^Eleven women had a missing value

^d^Twenty women had a missing value

^e^Three women had a missing value

^f^Forty women had a missing value

^g^Question was asked regarding the previous 30 day alcohol use in 2003–2004 but the previous 12 months in 2005–2010; 652 women did not answer this question

^h^Fourteen women had a missing value

### Excess adiposity and HPV infection

**Fig. 1:**
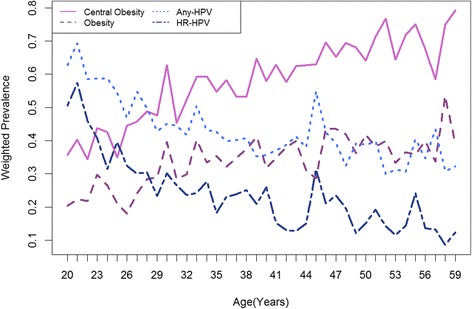
Age-specific prevalence of central obesity, obesity, any-type HPV and high risk (HR) type HPV in adult women enrolled in NHANES 2003–2010. Weighted prevalence estimates for women with central obesity, obesity, positive HPV DNA testing for any type or high-risk (or, oncogenic) types were displayed on the same graph. These single-year estimates for each age group were averages across the four survey cycles, despite of some variations, showing a generally increasing (for excessive adiposity) or decreasing trend (for HPV infection) towards older age. While these age-associated variations in central obesity and obesity parallel with each other, any-type and HR-type HPV prevalence became discordant around ages 40–44 years, at which time there was an apparent dip in HR-HPV prevalence

Women with central obesity had a slightly reduced (any-type: 41.1 % vs. 44.9 %, *P* = 0.045) or comparable (HR-type: 21.9 % vs. 25.4 %, *P* = 0.055) HPV prevalence. BMI-based adiposity measures, such as obesity (*P* = 0.451 for any-HPV; *P* = 0.191 for HR-HPV) and BMI categories (*P* = 0.36 for any-HPV; *P* = 0.345 for HR-HPV), were not associated with HPV prevalence (Table 1). Except for a diabetic history and current use of oral contraceptives, women who were positive for any-type and HR-type HPV DNA testing were likely to be young (Fig. 1) and shared common socio-demographic and behavioral risk factors (all *P*-values <0.001, Table 1). After adjusting for age, socio-economic factors and risky behaviors, centrally-obese women had a 9 % reduced prevalence for any-HPV (aPR = 0.91, 95 % confidence interval [CI] = 0.84–0.99; *P* = 0.03) but not for HR-HPV (aPR = 0.92, 95 % CI = 0.79-1.07; *P* = 0.279) infection as compared to women of normal WC (≤88 cm). Meanwhile, neither obesity nor a high waist-to-height ratio (>0.6) showed statistical associations with any-type or HR-type HPV infections (Table 2).

**Table 2:** Results of multivariable Poisson regression on any- or high risk-type HPV prevalence by central obesity or obesity, adult women aged 20–59 (*N* = 4126^a^) in NHANES 2003–2010

	Any-HPV				HR-HPV			
		95 % CI				95 % CI		
	aPR^b^	LL	UL	*p*-value	aPR^b^	LL	UL	*p*-value
WHtR > 0.6	0.98	0.90	1.07	0.671	0.95	0.84	1.08	0.437
Obesity (BMI ≥ 30 kg/m^2^)	0.94	0.86	1.02	0.109	0.90	0.79	1.02	0.097
Central obesity (waist >88 cm)	0.91	0.84	0.99	0.030	0.92	0.79	1.07	0.279

*BMI* body mass index. *CI* confidence interval, *aPR* adjusted prevalence ratio, *LL* lower limit, *UL* upper limit, *WHtR* Waist-to-height ratio

^a^Forty out of 4172 women had missing formation about number of lifetime partners; six were missing information about health insurance coverage

^b^Adjusting for survey cycle, age, race/ethnicity, college degree or not, marital status, health insurance coverage or not, and lifetime sexual exposure

In women who reported an early sex debut (<16 years), we noted that, obese women had 24 % lower likelihood for HR-HPV infection (aPR = 0.76, *P* = 0.017) than their counterparts whereas women who had sex debut at age 16 or later had a comparably low HR-HPV prevalence regardless of their BMI (*P*-value for interaction = 0.006, Table 3). Likewise, we also observed such differential effect on the association between central obesity and HR-HPV (*P*-value for interaction = 0.019, Table 3) by early sex debut. Despite of an overall null association between obesity or central obesity with any-type HPV infection, centrally-obese women were at a 13 % reduced probability for any-type HPV infection as compared to women with a normal WC (aPR = 0.87, *P* = 0.028) only in the non-Hispanic White subpopulation; though such racial differences were not statistically significant (Additional file 1: Figure S2).

**Table 3:** Results of stratified analysis by age first sex debut suggesting differential modulating effects the associations between excessive adiposity and risks for high-risk type HPV infection among adult women in NHANES 2003–2010 (*N* = 4124^a^)

HR-HPV	Reported age at first sex							
	<16 years (*n* = 1112)				> = 16 years (*n* = 3012)			
		95 % CI				95 % CI		
	aPR	LL	UL	*p*-value	aPR	LL	UL	*p*-value
Obesity vs. BMI < 30 kg/m^2^*	0.76	0.60	0.95	0.017	0.98	0.85	1.13	0.744
Central obesity vs. WC ≤ 88 cm**	0.72	0.59	0.89	0.003	1.03	0.86	1.24	0.726

*aPR* adjusted prevalence ratio, *BMI* body mass index, *CI* confidence interval, *HR* high risk type, *LL* lower limit, *NHANES* National Health and Nutrition Examination Survey, *UL* upper limit, *WC* waist circumference

*P for interaction = 0.006

**P for interaction = 0.019

^a^Forty out of 4172 women had missing formation about number of lifetime partners; six were missing information about health insurance coverage; two did not report age at sex debut

### Metabolic health and HPV infection

Among 1987 fasting women who were included for the subgroup analysis, prevalence of any-type (42.3 %) or oncogenic (22.9 %) HPV infection was comparable to that of the primary study population (Table 1). Despite that women of excess adiposity were similarly distributed in the fasting subgroup (Additional file 1: Table S1) as compared to the primary study population, fasting women who were positive for oncogenic HPV were more likely to be in the lean or normal-waist group (all *P*-values <0.05) in univariate analysis. Additionally, 57.9 % of fasting women were metabolically healthy with a BMI at the normal range, 7.7 % were metabolically unhealthy non-obese (MUNO), 16.0 % were metabolically healthy yet obese (MHO); 18.5 % met the ATP III criteria for metabolic syndrome and were obese. Prevalence of any-type HPV for women in the four metabolic health-obese groups was alike (*P* = 0.372) whereas that of oncogenic HPV was substantially lower in obese women with MetS (14.9 %, *P* = 0.005). Otherwise, distributions of fasting women with positive HPV DNA for any type or oncogenic types were also similar among different groups as compared to the primary population (Additional file 1: Table S2).

Results of multivariable Poisson regression showed that, in general, neither excessive adiposity nor metabolic health status was correlated with any-type HPV infection (Table 4). Nevertheless, obese women overall had a 23 % reduction in HR-HPV infection as compared to that of normal-BMI individuals (aPR = 0.77, *P* = 0.016; Table 4). Metabolically unhealthy obese women also had a more than 30 % lower probability for HR-HPV infection than women with normal BMI and metabolic health status did (aPR = 0.69, *P* = 0.018; Table 4). We did not identify any significant host characteristics that moderated fasting women’s likelihood for HPV infection in further subgroup analysis.

**Table 4:** Results of multivariable Poisson regression on any- or high risk-type HPV prevalence by adiposity and metabolic health status, fasting adult women aged 20–59 (*N* = 1972^a^) in NHANES 2003–2010

	Any-HPV				HR-HPV			
	aPR^b^	95 % CI			aPR^b^	95 % CI		
		LL	UL	*p*-value		LL	UL	*p*-value
WHtR > 0.6	0.97	0.86	1.08	0.542	0.82	0.66	1.02	0.074
Obesity (BMI ≥ 30 kg/m^2^)	0.96	0.85	1.09	0.534	0.77	0.62	0.95	0.016
Central obesity (WC >88 cm)	0.96	0.86	1.08	0.502	0.89	0.72	1.09	0.254
MetS	0.98	0.84	1.14	0.760	0.85	0.68	1.08	0.176
Metabolic health and obesity								
Normal	1.00				1.00			
MUNO	1.06	0.83	1.36	0.625	1.19	0.88	1.62	0.263
MHO	1.00	0.84	1.19	0.971	0.86	0.68	1.09	0.204
MetS & obese	0.94	0.80	1.11	0.478	0.69	0.51	0.94	0.018

*aPR* adjusted prevalence ratio, *BMI* body mass index, *CI* confidence interval, *LL* lower limit, *MetS* metabolic syndrome by ATP III criteria, *MHO* metabolically healthy and obese, *MUNO* metabolically unhealthy and non-obese, *UL* upper limit, *WC* waist circumference, *WHtR* waist-to-height ratio

^a^Fifteen out of 1987 women had missing formation about either number of lifetime partners or health insurance coverage

^b^Adjusting for survey cycle, age, race/ethnicity, college degree or not, marital status, health insurance coverage or not, and lifetime sexual exposure

## Discussion

The current study extended a previous investigation on the obesity-HPV association from a specific age group (aged 35–60) [12] to adult women in general (aged 20–59). Overall, we found a null or weak association between obesity or central obesity and HPV infection in this nationally-representative adult female population. However, subgroup analysis showed that obese or centrally-obese women had a significantly reduced HR-HPV infection among those who reported an early sex debut (<16 years) or those included in the fasting subpopulation. We proposed three potential mechanisms for these observed negative correlations.

First, despite the early sex debut, adult women with excessive adiposity might have a lower HPV burden than women with normal BMI or WC. Early studies on adolescents have consistently linked early sex debut to an increased risk for sexually transmitted infections (STI), including HPV infection [30, 31]. The heightened STI risks were reportedly mediated by concomitant risky behaviors such as frequency of sex, irregular use of condom, alcohol use and a smoking habit [31]. In our analysis, additional adjustment for behavioral factors, such as condom use, drinking history in the last 12 months and cumulative smoking exposure did not change our results. It was possible that obese or centrally obese women who initiated sex activities early may have had a less risky behavioral profile, either in adolescence or early adulthood, resulting in fewer new acquisitions or re-infections overall.

Secondly, we hypothesized that the altered host immune responses in obese or centrally-obese women may have facilitated viral clearance (in adolescence) or suppress viral reactivation from latent, persistent HPV infections (in mid- or late adulthood). Higher concentrations of several cytokines, including tumor necrosis factor (TNF)-α, interleukin (IL)-6 and others, were found in immature cervical epithelium than those in mature, squamous cervical epithelium [32]. Whether such elevation in innate immune responses translates into effective defenses against viral pathogens in adolescents remains unknown. Women who had been obese (or centrally-obese) since adolescence could have outperformed their peers in controlling early HPV infections due to their pro-inflammation milieu. Besides, for adult women with excessive adiposity, their hyper-activated adaptive immunity [33] might have held latent viruses in check, reducing chances of ageing-associated viral reactivation as compared to women of normal BMI or WC [34].

Lastly, obese individuals may have paid more visits to healthcare providers because of their cardio-metabolic co-morbidities, such as hypertension or diabetes [12]. The observed negative associations between excessive adiposity and HR-HPV infection were probable results of vigilant screening or diagnosis. Furthermore, a cohort effect could have also explained for an overall, low lifetime exposure risk for HPV in older women; [35] this potential risk-modifying effect by age at sex debut was particularly evident in the younger (aged 20–34) than the older cohorts (aged 35–50 or 50–59).

To our knowledge, the current study was also among the few investigations in examining metabolic health effects on a specific infection. The finding that obese or MUO (metabolically unhealthy obese) women showed a significantly low probability for HR-HPV but not any-type HPV infection has raised our concerns about a biased, fasting subpopulation. Statistically, we have accounted for the relative low participation of diabetic participants in the fasting session by use of appropriate sampling weights as suggested [22]. Fasting women also appeared to have a similar degree of adiposity to those in the primary analysis in terms of BMI, waist circumference or WHtR (Additional file 1: Table S1). The only apparent difference between the primary and the fasting population was the highest degree of educational attainment by study participants; 29.7 % of fasting women reported a college education or higher whereas 26.2 % of the overall analytic sample did so (*P* = 0.041). We believe that selection bias was unlikely to fully explain for the strong reverse associations found between HR-HPV and metabolically unhealthy obesity or obesity alone. Large longitudinal studies are needed to prospectively assess influences of excessive adiposity and its associated metabolic derangements on the natural history of HPV infection.

### Limitations

There are several limitations in interpreting our study results. First, the lack of temporality was inherent in the cross-sectional data. Cervical HPV infection is a well-defined local infection, and the potential for causing systemic ectopic disposition of fatty tissue is thus unlikely. Nevertheless, the conjecture of infection-associated adipogenesis needs to be prospectively evaluated as in other obesity-inducing viral infections [36]. Secondly, the current study could not fully examine whether or how the HPV immunization could have affected the adiposity-HPV relationship. The documentation of HPV vaccination among survey participants began since 2006 and only a minority of women had received the vaccine: 3.1 % in 2007–2008 and 5.2 % in 2009–2010. When we repeated the analysis on women enrolled in these two survey cycles (2007–2010, *N* = 2568) and adjusted for the HPV vaccination history, the strength of association between central obesity and any-HPV was similar (aPR = 0.92) but was not statistically significant (*P* = 0.171). Moreover, as we did not include women of BMI < 18.5 Kg/m^2^ in the current analysis, whether low adiposity correlated with a disproportionately high likelihood for prevalent HPV (supporting a monotonically deceasing, dose–response association); or whether women of an abnormally low BMI they similarly exhibited low rates of HPV infection (suggesting an inverse U-shaped association) remains unexplored.

The current study also failed to examine for the contribution of inflammatory proteins or cytokines to the observed reverse associations because conventional inflammatory biomarkers, including ferritin or C-reactive protein, were not available for women of all age groups [21]. Among well-studied adipokines secreted by white adipose tissues, leptin and adiponectin have been implicated in changed immune responses by exerting their pro- and anti-inflammatory activity in both innate and adaptive immunity, separately [37, 38]. To elucidate the mechanism involved in the polarization between pro- and anti-inflammatory pathways during the development and maintenance of visceral adiposity, longitudinally collected immune correlates at the cervix may provide anchoring information to disentangle the causal from collateral events.

Lastly, according to Sumner and colleagues, the currently-adopted, lipid-based definition for MetS were likely to misclassify NHB women as metabolically healthy when in fact they might already exhibit significant insulin resistance [39]. Yet, ad-hoc sensitivity analysis that excluded NHB women from the fasting population revealed unaltered results (data not shown). As such, we believed that findings of the current study were at little risk for ethnicity-associated misclassification bias.

## Conclusions

In this US-representative adult female population, obesity or central obesity was not substantially associated with HPV infection in general. However, in certain subpopulations, women with excessive adiposity or its relevant metabolic dysfunction showed a considerably reduced HR-HPV prevalence. Particularly, early sex debut in adolescence seemed to modulate such reduction in obese or centrally-obese individuals. Prospective studies are needed to further assess the influence of excessive adiposity on the natural course of HPV infection, particularly on the initial acquisition and clearance in early adulthood as well as on the viral persistence in later adulthood.

## Additional file

Additional file 1: Figures S1-2 and Tables S1-2. (DOCX 671 kb)
