# Supplementary material for: Excessive adiposity, metabolic health, and risks for genital human papillomavirus infection in adult women: a population-based cross-sectional study
Source: BMC Obes. 2015 Oct 1;2:39. doi: 10.1186/s40608-015-0071-3 (PMC4591625; doi:10.1186/s40608-015-0071-3)
Supplement: Additional file 1: Figures S1-2 and Tables S1-2. — (DOCX 671 kb) [file 40608_2015_71_MOESM1_ESM.docx]

Supplementary Material for *BMC Women’s Health*

**Title:** Excessive adiposity, metabolic health, and risks for genital human papillomavirus infection in adult women: a population-based cross-sectional study

**Authors:** S. H. Liu, H. J. Chen, T. H. Hsieh, J. C. Chen, Y. C. Huang

**Content:** Supplementary Figures S1-2 and Tables S1-2

**Supplementary Figure S1**


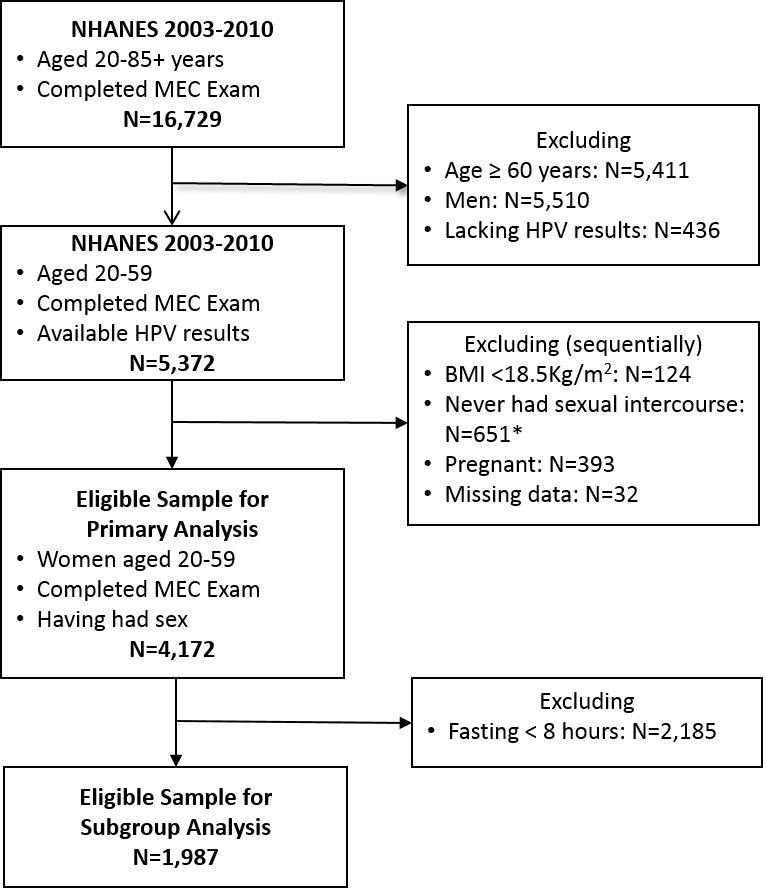


**Supplementary Figure S2**

**
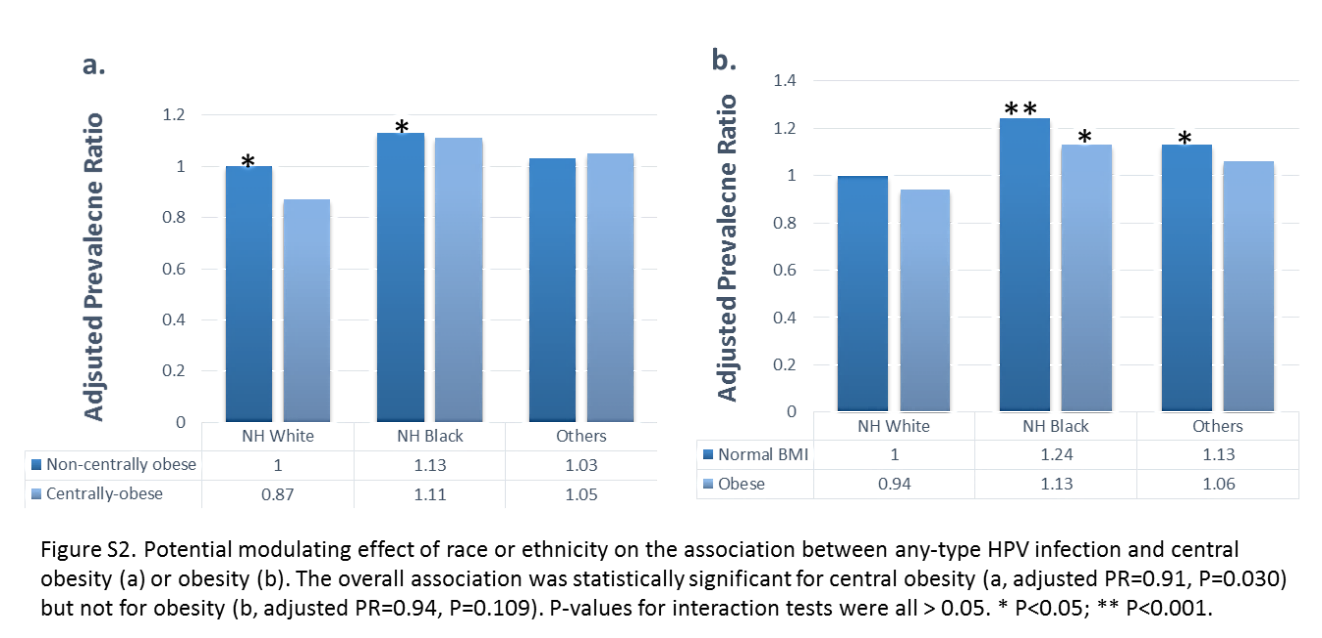
**

**Supplementary Table S1**

| **Table S1**. Comparison of the primary study population and the fasting subgroup by selected characteristics, NHANES 2003-2010 | | | |
| --- | --- | --- | --- |
| Characteristics (weighted %) | Primary (N=4172) | Fasting (N=1987) | p-value |
| Central obesity: waist > 88cm | 58.0% | 60.3% | 0.183 |
| Obesity: BMI >= 30Kg/m2 | 34.3% | 34.4% | 0.975 |
| Waist-to-height ratio: ≥ 0.6 | 35.9% | 36.0% | 0.933 |
| BMI, Kg/m^2^ |  |  |  |
| 18.5-25 | 37.9% | 37.1% | 0.929 |
| 25-30 | 27.7% | 28.5% |  |
| 30-35 | 17.0% | 17.3% |  |
| ≥ 35 | 17.4% | 17.1% |  |
| Education: >=College | 26.2% | 29.7% | 0.041 |
| Hypertension history | 11.7% | 11.1% | 0.431 |
| Diabetes history | 3.1% | 2.7% | 0.803 |
| Age at sex debut >=16 years | 74.8% | 74.4% | 0.819 |
| Use of birth control pills: currently | 11.7% | 12.9% | 0.368 |

| **Table S2.** Weighted prevalence of any-type and high-risk type HPV by selected characteristics of fasting women aged 20-59 (N=1987) in NHANES 2003-2010 | | | | | |
| --- | --- | --- | --- | --- | --- |
| **Participant characteristics** | Any-type HPV | |  | High-risk type HPV | |
|  | % | p-value^a^ |  | % | p-value^a^ |
| **Overall** | 42.3% |  |  | 22.9% |  |
| **Survey year** |  |  |  |  |  |
| 2003-2004 | 48.8% | 0.156 |  | 24.7% | 0.698 |
| 2005-2006 | 39.2% |  |  | 24.0% |  |
| 2007-2007 | 40.6% |  |  | 20.7% |  |
| 2009-2010 | 41.5% |  |  | 22.8% |  |
| **Adiposity metrics** |  |  |  |  |  |
| Central obesity: waist > 88cm | 41.3% | 0.231 |  | 21.0% | 0.033 |
| waist <= 88cm | 43.8% |  |  | 25.9% |  |
| Obesity: BMI >= 30Kg/m2 | 42.0% | 0.863 |  | 19.1% | 0.021 |
| BMI < 30 Kg/m2 | 42.5% |  |  | 24.9% |  |
| Waist-to-height ratio: ≥ 0.6 | 41.3% | 0.540 |  | 19.4% | 0.020 |
| < 0.6 | 42.9% |  |  | 24.9% |  |
| BMI, Kg/m^2^ |  |  |  |  |  |
| 18.5-25 | 40.9% | 0.417 |  | 23.0% | 0.012 |
| 25-30 | 44.6% |  |  | 27.4% |  |
| 30-35 | 39.8% |  |  | 16.8% |  |
| ≥ 35 | 44.1% |  |  | 21.5% |  |
| **Metabolic Health** |  |  |  |  |  |
| Metabolically healthy & non-obese | 42.6% | 0.372 |  | 25.2% | 0.005 |
| Metabolically unhealthy & non-obese | 41.5% |  |  | 22.7% |  |
| Metabolically healthy & obese | 46.4% |  |  | 24.0% |  |
| Metabolically unhealthy & obese | 38.2% |  |  | 14.9% |  |
| **Socio-demographics** |  |  |  |  |  |
| Age, years |  |  |  |  |  |
| 20-34 | 50.7% | <0.001 |  | 32.6% | <0.001 |
| 35-49 | 41.4% |  |  | 21.1% |  |
| 50-59 | 32.7% |  |  | 12.9% |  |
| Race/ethnicity |  |  |  |  |  |
| Non-Hispanic White | 38.7% | <0.001 |  | 20.9% | 0.006 |
| Non-Hispanic Black | 58.5% |  |  | 30.3% |  |
| Mexican American/others | 46.3% |  |  | 26.0% |  |
| Education ≥ College degree | 33.5% | <0.001 |  | 16.1% | <0.001 |
| High school or less | 46.0% |  |  | 25.8% |  |
| Marital status |  |  |  |  |  |
| Married | 29.1% | <0.001 |  | 12.2% | <0.001 |
| Single | 59.0% |  |  | 35.8% |  |
| Living with a partner | 61.2% |  |  | 40.4% |  |
| Health insurance coverage^b^: yes | 39.8% | <0.001 |  | 21.2% | 0.001 |
| no | 53.1% |  |  | 30.8% |  |
| **Behavioral risks** |  |  |  |  |  |
| Hypertension history^c^: yes | 41.3% | 0.729 |  | 19.1% | 0.055 |
| no | 42.4% |  |  | 24.0% |  |
| Diabetes history^d^: yes | 56.3% | 0.034 |  | 20.1% | 0.597 |
| no | 41.5% |  |  | 23.1% |  |
| Lifetime smoking: ≥ 100 cigarettes | 47.0% | 0.013 |  | 24.3% | 0.368 |
| < 100 cigarettes | 38.8% |  |  | 21.8% |  |
| Last 12 months had ≥ 12 alcohol drinks^e^ | 43.8% | 0.038 |  | 24.7% | 0.006 |
| < 12 alcohol drinks | 38.1% |  |  | 17.6% |  |
| Age at sex debut >=16 | 38.8% | <0.001 |  | 21.1% | 0.007 |
| <16 | 52.7% |  |  | 28.1% |  |
| Number of lifetime sex partners >=5^f^ | 53.0% | <0.001 |  | 30.1% | <0.001 |
| <5 | 28.2% |  |  | 13.6% |  |
| Condom use: not always^g^ | 52.6% | <0.001 |  | 32.1% | <0.001 |
| always | 36.6% |  |  | 18.6% |  |
| Use of birth control pills: currently^h^ | 44.5% | 0.604 |  | 28.9% | 0.091 |
| not currently | 42.1% |  |  | 22.0% |  |
| Abbreviations: BMI, body mass index; SE, standard error | | | | | |
| a. P-value for survey-based Chi-squared test | |  |  |  |  |
| b. Six women had a missing value |  |  |  |  |  |
| c. Seven women had a missing value |  |  |  |  |  |
| d. Twenty women had a missing value |  |  |  |  |  |
| e. Three women had a missing value |  |  |  |  |  |
| f. Forty women had a missing value |  |  |  |  |  |
| g. Question was asked regarding the previous 30 day alcohol use in 2003-2004 but the previous 12 months in 2005-2010; 652 women did not answer this question | | | | | |
